# Temporal gene expression profiling of the rat knee joint capsule during immobilization-induced joint contractures

**DOI:** 10.1186/s12891-015-0588-0

**Published:** 2015-05-26

**Authors:** Kayleigh Wong, Fangui Sun, Guy Trudel, Paola Sebastiani, Odette Laneuville

**Affiliations:** Department of Biochemistry, Microbiology and Immunology, Faculty of Medicine, University of Ottawa, 451 Smyth Rd, Ottawa, ON K1H 8M5 Canada; Department of Biostatistics, Boston University School of Public Health, Medical Campus, 801 Massachusetts Ave., Crosstown 3rd floor, Boston, MA 02118 USA; The Ottawa Hospital Rehabilitation Centre, 505 Smyth Rd., Ottawa, ON K1H 8M2 Canada; Bone and Joint Research Laboratory, Faculty of Medicine, 451 Smyth Rd., Ottawa, ON K1H 8M5 Canada; Department of Biology, Faculty of Science, University of Ottawa, 30 Marie Curie, Ottawa, ON K1N 6N5 Canada

**Keywords:** Joint contracture, Immobilization, Knee joint capsule, Gene expression, Rat

## Abstract

**Background:**

Contractures of the knee joint cause disability and handicap. Recovering range of motion is recognized by arthritic patients as their preference for improved health outcome secondary only to pain management. Clinical and experimental studies provide evidence that the posterior knee capsule prevents the knee from achieving full extension. This study was undertaken to investigate the dynamic changes of the joint capsule transcriptome during the progression of knee joint contractures induced by immobilization. We performed a microarray analysis of genes expressed in the posterior knee joint capsule following induction of a flexion contracture by rigidly immobilizing the rat knee joint over a time-course of 16 weeks. Fold changes of expression values were measured and co-expressed genes were identified by clustering based on time-series analysis. Genes associated with immobilization were further analyzed to reveal pathways and biological significance and validated by immunohistochemistry on sagittal sections of knee joints.

**Results:**

Changes in expression with a minimum of 1.5 fold changes were dominated by a decrease in expression for 7732 probe sets occurring at week 8 while the expression of 2251 probe sets increased. Clusters of genes with similar profiles of expression included a total of 162 genes displaying at least a 2 fold change compared to week 1. Functional analysis revealed ontology categories corresponding to triglyceride metabolism, extracellular matrix and muscle contraction. The altered expression of selected genes involved in the triglyceride biosynthesis pathway; AGPAT-9, and of the genes P4HB and HSP47, both involved in collagen synthesis, was confirmed by immunohistochemistry.

**Conclusions:**

Gene expression in the knee joint capsule was sensitive to joint immobility and provided insights into molecular mechanisms relevant to the pathophysiology of knee flexion contractures. Capsule responses to immobilization was dynamic and characterized by modulation of at least three reaction pathways; down regulation of triglyceride biosynthesis, alteration of extracellular matrix degradation and muscle contraction gene expression. The posterior knee capsule may deploy tissue-specific patterns of mRNA regulatory responses to immobilization. The identification of altered expression of genes and biochemical pathways in the joint capsule provides potential targets for the therapy of knee flexion contractures.

## Background

Many joint diseases, including both inflammatory and non-inflammatory types, are associated with a reduced mobility of the affected joints. The inability of the joint to range through its full passive motion is referred to as a contracture, the nature of which is chronic and treated with limited success [1, 2]. For rheumatoid arthritis (RA) patients, the lack of complete joint mobility is the second most troublesome problem after pain and results in functional impairment and limitation of activities [3]. Consistently, the second preference for improvement of health for RA patients is mobility with emphasis on walking and handling activities [4]. Knee contractures are also a complication of arthroplasty (TKA) preventing the knee joint from achieving the full extension that is essential for normal walking [5–7]. Postoperative manual stretching aims at restoring the loss of range of motion (ROM) but pain and the high demands of this prolonged rehabilitation treatment can hamper the efforts and patients may become disabled [8]. Revision surgery can provide temporary improvement in some cases but the risk of postsurgical complications is high [9]. Overall, joint contractures have limited reversibility, permanently impairing the physical ability of individuals.

The etiology of joint contractures, whether in association with arthritic diseases or not, remains unknown and few studies have been conducted to identify causal factors. A retrospective review of data collected from patients undergoing primary TKA reported a 3.6 % incidence of flexion contractures at 2 years postoperative [6]. Although at low percentage, more than 4630 new patients develop flexion contractures after TKA every year in the United States based on the 130 000 knee replacements performed for patients with disabling arthritis of the knee. The two most important contributors to flexion contractures following primary TKA were identified as bone deformity and soft tissue tightness [6, 8]. In a different clinical context, joint contractures were prevalent in patients admitted to intensive care units (ICU) and with a stay longer than 2 weeks [10]. A 34 % incidence of joint contracture in a large joint was reported indicating that immobility was an important etiologic factor for joint contractures. Time spent in the ICU was a significant risk factor for contracture; a stay of 8 weeks or longer was associated with a significantly greater risk of any joint contracture than a stay of 2 to 3 weeks [11]. An animal model of joint contractures was developed to evaluate the causal role of prolonged joint immobility and to study the pathophysiology involved [12]. Both clinical and experimental evidence support the notion of immobilization causing joint contractures over time.

The contribution of time in the initiation, progression, and severity of joint contractures induced by immobilization was confirmed and the loss of ROM of rat knees immobilized in flexion over a 32-week time course that was gradual over the first 8 weeks and then reached a plateau [12]. The immobilization device was implanted outside the joint to preserve the integrity of joint tissue structures and prevented the knee from achieving full extension. Upon removal of the immobilization device, the ROM was measured and results indicated that a fixed flexion contracture was present [12, 13]. Sectioning of the posterior knee joint muscles did not restore the full ROM and limitation was attributed to the capsule [14]. Histological analysis of knee sections corroborated the biomechanical limitation mediated by the posterior capsule and a reduction of the synovial length was measured [15]. The progressive loss of the knee ROM over time was indicative of a dynamic process leading to structural changes of the capsule tissue and the next step was to identify the molecular events involved.

The aim of the current study was to establish the dynamic profile of gene expression in the posterior knee capsule tissue over the course of the development of a joint contracture induced by immobilization.

## Results

From the initial 80 rats operated, two were eliminated because of a wound infection. Extracted RNA samples from the remaining 78 posterior knee capsules were submitted to quality control analysis and 20 sham-operated and 20 immobilized samples were selected for microarray analysis on the basis of similar RNA yield ranging between 50 and 80 ng/μl.

### Fold changes in gene expression

**Fig. 1:**
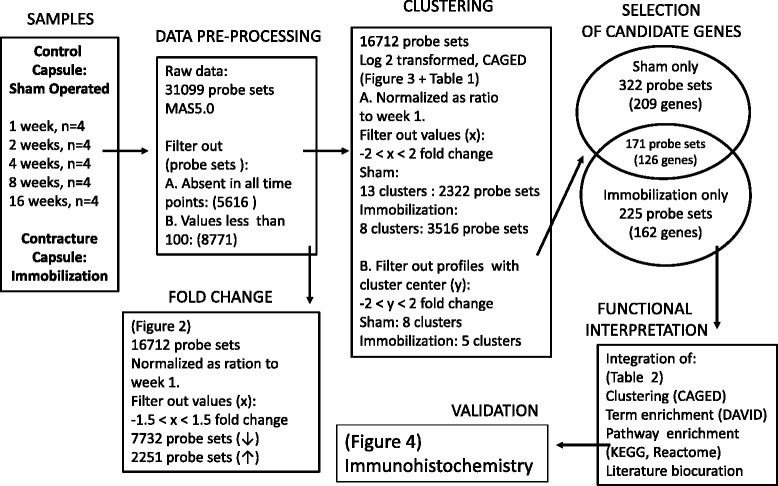
Experimental design to identify capsule genes associated with immobilization-induced joint contractures. After preprocessing raw microarray data, intensity values of probe sets measured at 2, 4, 8, and 16 weeks were compared to results measured at week 1 to determine the fold change in expression. Probe sets counts were separated into four bins: up-regulated by a fold change of 1.5 to 2.0 or above 2.0, and down-regulated by a fold change of −1.5 to −2 or more than −2. The number of probe sets included in each bin is indicated on the Y axis for individual time points indicated on the X axis

**Fig. 2:**
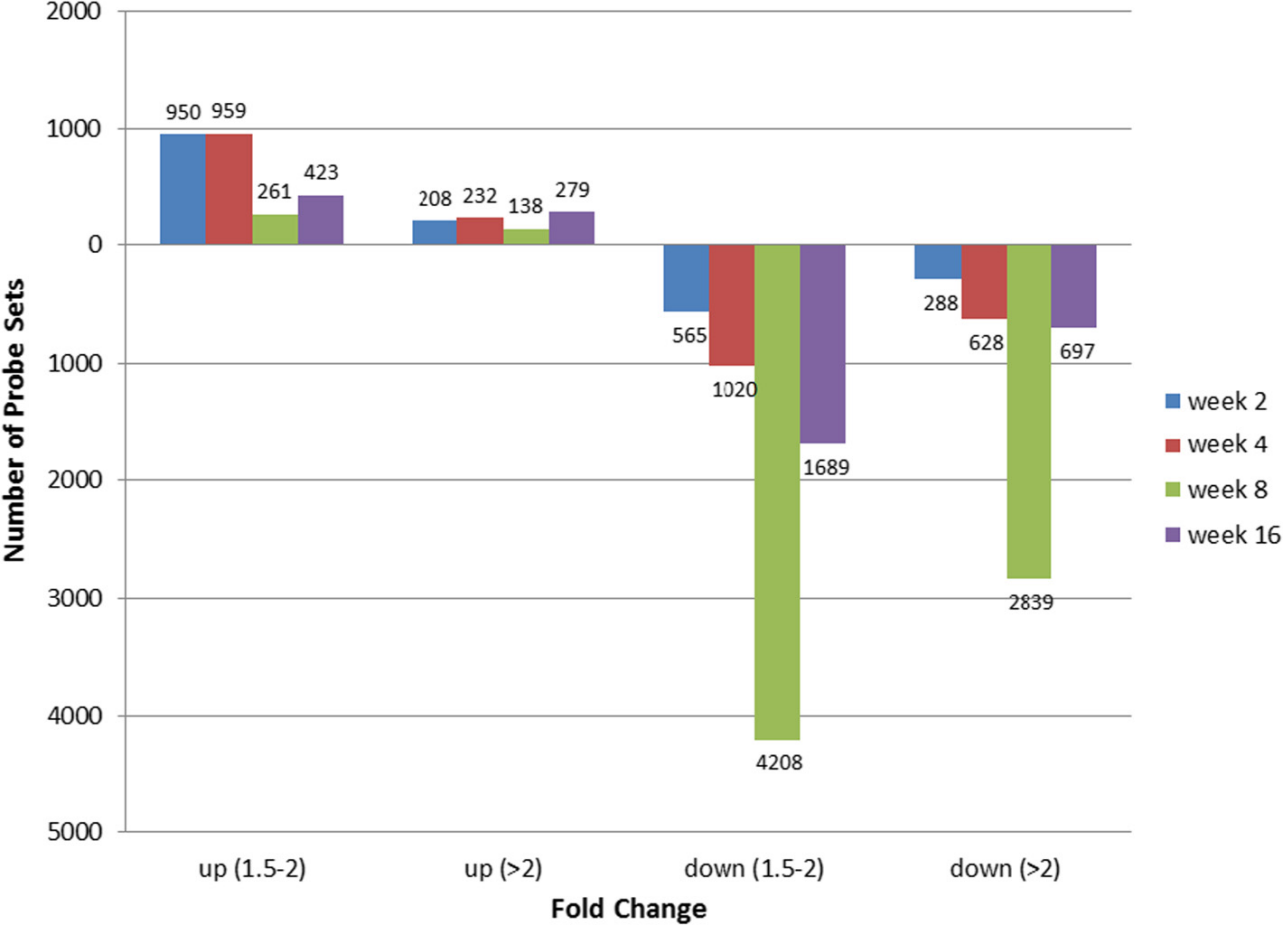
Fold changes in gene expression in the capsule of immobilized knee joints. After pre-processing signal intensities from the immobilization group, expression levels at 2, 4, 8, and 16 weeks were log 2 transformed and values compared to the log 2 transformed results at week 1 using the CAGED algorithm to cluster genes with similar profiles of expression. Eight expression clusters were identified and clusters 2 and 3 had median fold changes between −2 and 2 and were thus eliminated. For the remaining 6 clusters, cluster centers corresponding to log2 value over the time course of immobilization-induced knee joint contracture were plotted over the time-course

The complete data sets of pre-processed data included expression values for 16712 probe sets measured in both sham-operated and immobilized knee capsules (Fig. 1). To appreciate fold changes in gene expression, week 1 was set as the reference point and ratios were analyzed independently for each series. Ratios with a minimum of a 1.5 fold change were binned in two categories; 1.5 to 2.0 and 2.0 more than. The direction and magnitude of fold changes in expression were dominated by a reduction in expression for 7732 probe sets compared to 2251 probe sets increasing (Fig. 2). Expression changes were time dependent; a decrease occurred more frequently at week 8 for 92.5 % (=7141/7732 probe sets) of the total probe sets decreasing at any time point. Increase in expression was observed at week 2 and 4 for 1736 probe sets or 77.1 % (1736/2251).

### Time-related patterns of gene expression

**Fig. 3:**
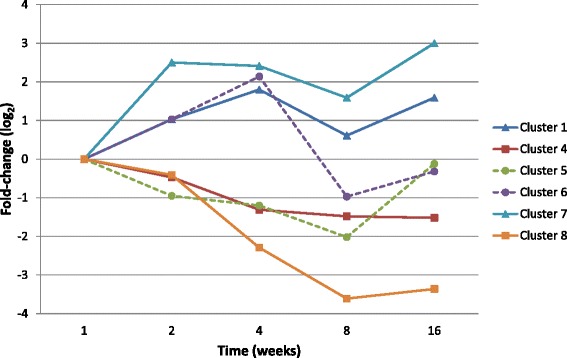
Time-series analysis to identify clusters of expression associated with immobilization. Posterior knee capsule samples from sham-operated and immobilized rats joints were harvested at time points of 1, 2, 4, 8, and 16 weeks and were subjected to genome-wide microarray analysis. Expression values from the sham and immobilized samples were analyzed separately to quantify fold changes and to cluster genes with similar profiles of expression. The two lists of genes included in clusters generated from temporal expression analysis were compared to identify similarities. Genes unique to immobilization were analyzed in depth to gain insight on the biological significance by integration of CAGED, GO, KEGG, Reactome, and literature biocuration results. Immunohistochemistry was used to validate the microarray results for selected genes

To capture gene expression dynamics, CAGED algorithm was applied to log 2 transformed data included in the complete data sets. Probe sets with at least one time point observation above or below 2 fold change compared to week 1, were considered in the clustering analysis and summed to a total of 2322 for the sham operated group and to 3516 for the immobilization group (Fig. 1). Clustering of sham-operated data identified 13 clusters each including genes with a similar profile of expression. Eight clusters with center values, in log 2 base, higher than 1 or lower than −1, were selected for in depth analysis. The total number of probe sets included in the eight sham-operated clusters was equal to 493 corresponding to 335 different genes. Cluster analysis of the immobilization gene-expression time series identified 8 distinct clusters. Cluster center values were used to select clusters for in depth analysis and additional grouping. Six clusters were assigned to one of the three major temporal groups according to increased (clusters 1 and 7), variable (clusters 5 and 6), or decreased expression (clusters 4 and 8) (Fig. 3). The total number of probe sets included the 6 clusters of the immobility group was 396 corresponding to 288 genes.

### Selection of capsule immobilization genes

The list of probes sets included in all selected clusters from sham-operated and immobilized samples were compared to identify similarities (Fig. 1) and 171 probe sets, corresponding to 126 genes, were found in both groups. Identified genes with a profile of differential expression common to both groups; immobilization and sham-operated, although not specific to knee flexion contractures, could play a role in the capsular changes leading to a restriction of knee ROM induced by immobility but were not further studied. The number of probe sets included in the immobilization group only was equal to 225 corresponding to 162 different genes that were further analyzed to obtain biological insight. The aim here was to study the temporal changes in gene expression associated with immobilization and a total of 162 genes displayed dynamic changes of expression associated with the progression of knee joint contracture over the time-course of 16 weeks.

### Functional knowledge base

The list of 162 immobilization genes included in selected clusters was analyzed for enrichment of biological functions. A GO enrichment analysis was conducted using DAVID/EASE (version 6.7) software and Affymetrix probe ID notations. Immobilization genes were classified into three different categories belonging to Biological Process, Molecular Function and Cellular Component. The two most represented GO terms were: extracellular matrix structure and organization and lipid biosynthetic and metabolic process. Immobilization genes included in GO terms groups were compared to the list of genes included in clusters generated by CAGED analysis (Table 1). Interestingly, the majority of the genes included in the most represented GO terms belonged to cluster 4 from CAGED analysis; 24 of 36 (67 %) genes for the Biological Process and 36 of 63 (57 %) genes for Cellular Component.

**Table 1:** Functional interpretation of genes associated with immobilization-induced knee joint contractures. The lists of genes included in all clusters identified for the sham-operated and for the immobilization group were compared for similarities. Genes associated with the immobilization intervention only were submitted to Gene Ontology (GO), KEGG Pathways, and Reactome Pathway analysis. Analyses were performed using default settings for terms of the GO Biological Processes (BP_FAT), GO Molecular Function categories (MF_FAT) and GO Cellular Component (CC_FAT) (not shown). Significant GO terms and pathways from KEGG and Reactome mapping are listed. The list of genes included in each enriched terms was compared to immobilization genes identified by clustering using CAGED application. Numbers in parenthesis indicate the number of common probe sets between enriched terms and CAGED clusters

GO Terms	P-value	CAGED cluster (# of probes in cluster)	KEGG Pathways	P-value	CAGED cluster (# of probes in cluster)	Reactome Pathways	P-value	CAGED cluster (# of probes in cluster)
**Biological Process**			Dilated cardiomyopathy	4.8 x 10^−4^	1 (2)	Muscle contraction	5.0 x 10^−10^	1 (3)
					4 (3)			4 (3)
					5 (4)			5 (4)
					8 (1)			8 (1)
**Extracellular structure organization**	9.2 x 10^−7^	1 (5)	ECM-receptor interaction	2.0 x 10^−3^	1 (2)	Triglyceride biosynthesis	3.6 x 10^−7^	4 (9)
		4 (4)			4 (1)			
		5 (1)			6 (3)			
		6 (2)			8 (1)			
**Lipid biosynthetic process**	1.9 x 10^−5^	1 (3)	Hypertrophic cardiomyopathy (HCM)	2.4 x 10^−3^	1 (2)	Gelatin degradation by MMPs	3.8 x 10^−7^	1 (2)
		4 (12)			4 (2)			4 (3)
					5 (4)			6 (2)
					8 (1)			
**Neutral lipid metabolic process**	2.0 x 10^−5^	1 (1)	Cardiac muscle contraction	1.1 x 10^−2^	4 (2)	Collagen type XI degradation by MMPs	9.7 x 10^−7^	1 (2)
		4 (8)			5 (4)			4 (2)
					8 (1)			6 (1)
**Cellular Component**			Focal adhesion	2.2 x 10^−2^	1 (3)	Degradation of collagen	1.3 x 10^−6^	1 (2)
					4 (1)			
					6 (3)			4 (5)
					8 (1)			6 (2)
**Extracellular region part**	8.6 x 10^−9^	1 (7)	Biosynthesis of unsaturated fatty acids	2.9 x 10^−2^	4 (3)	Collagen type X degradation by MMPs	2.2 x 10^−6^	4 (2)
		4 (15)						6 (1)
		5 (1)						
		6 (4)						
		8 (1)						
**Extracellular matrix**	2.0 x 10^−8^	1 (4)	Complement and coagulation cascades	4.4 x 10^−2^	4 (3)	ATP Hydrolysis by Myosin; Myosin Binds ATP; Calcium Binds Caldesmon; Release of ADP From Myosin	4.8 x 10^−6^	1 (3)
		4 (9)			5 (1)			4 (1)
		6 (4)						5 (2)
		8 (1)						8 (1)
**Proteinaceous extracellular matrix**	2.2 x 10^−8^	1 (4)	PPAR signalling pathway	4.5 x 10^−2^	4 (5)	Degradation of the extracellular matrix	7.5 x 10^−6^	1 (1)
		4 (8)						4 (5)
		6 (4)						6 (2)
		8 (1)						
			Glycine, serine and threonine metabolism	5.0 x 10^−2^	1 (1)	Smooth Muscle Contraction	7.9 x 10^−6^	1 (3)
					4 (2)			4 (1)
								5 (2)
								8 (1)

The immobilization genes were mapped to their corresponding metabolic pathways using KEGG pathway analysis and the Reactome software. Most represented KEGG pathways included dilated cardiomyopathy, extracellular matrix, and biosynthesis of fatty acids (Table 1). Genes included in the most represented KEGG pathways were compared to the genes identified by clustering and the majority, 36 of 63 (=57 %) genes, belonged to cluster 4 (Table 1). The most represented Reactome pathways included: muscle contraction, triglyceride biosynthesis, and collagen degradation (Table 1). The list of genes included in the most represented Reactome pathways was compared to clustering results and the majority, 31 of the 56 genes or 55 %, belonged to CAGED cluster 4.

### Genes and pathways of the capsule response to immobilization

The list of enriched genes related to the biosynthesis of triglycerides included: *Fasn, Agpat2, Ggat2, Acaca, Gpd1, Elov1,* and *Acsl1*. All seven genes displayed a reduction in expression over the immobilization time course and belonged to cluster 4 of CAGED analysis. For the biological term extracellular structure and degradation, genes with an altered profile of expression included: *MMP3, MMP9, MMP13, Col2a1, Col10a1, Col11a1, Agt, Alb, Cdh1, Cdh2, Cfd, Chad, Ibsp, Ky, Myh11, Obp3, Pcsk6, Tf, Tnfrsf11b, Tnn,* and *Vit*. Of particular interest for the capsule tissue are the metalloproteinase genes (MMP), all displaying a profile of decreased expression during the progression of the knee contracture. The collagen genes enriched in the immobilization group displayed either variable or reduced expression over the time-course.

### Human capsule gene enrichment analysis: literature searching

Available data sources were searched to build a target network to gain further understanding of the gene expression changes taking place in the knee capsule of contractured joints. To our knowledge, no published literature or data deposited in public databases on genome-wide expression of the knee joint capsule tissue is currently available. An enrichment analysis for human joint diseases characterized by the presence of either joint contractures or joint hyper laxity was established and included 122 genes. Results from the enrichment analysis were compared to the list of genes generated from our study on immobilization-induced knee joint contractures and common genes included: *Col2a1, Col10a1, Col11a1, TNNT1, TNNT2, TPM3,* and *PLP1*.

### Immunohistochemistry validation

**Fig. 4:**
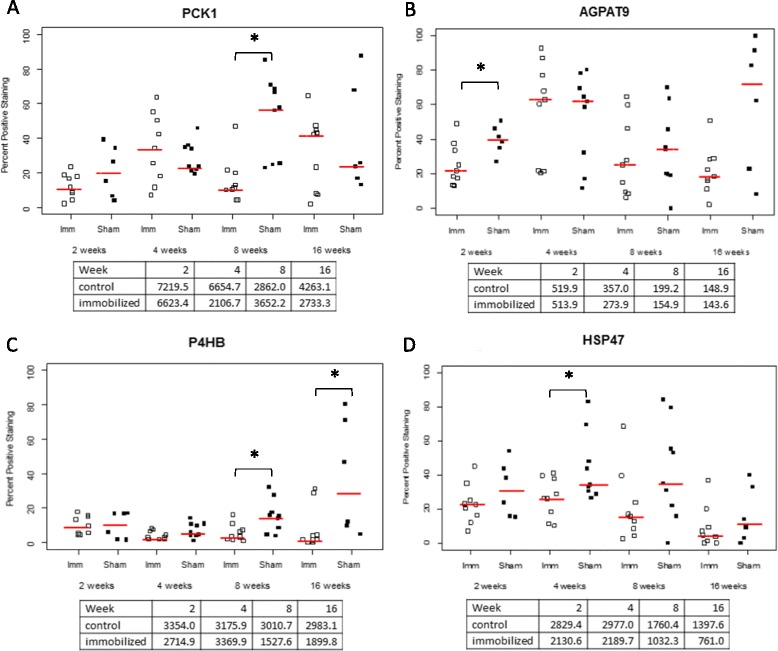
Validation of immobilization associated genes: immunohistochemistry. Quantification of immunohistochemistry staining of PCK1 (panel A), AGPAT-9 (panel B), P4HB (panel C), and HSP47 (panel D) in the posterior knee capsule from the rat time course experiment. The average count of each field between two independent examiners is plotted along with a median line for each experimental group. Statistical significance (p < 0.05) in levels of protein staining is indicated with*. Values below individual graphs represent pre-processed median mRNA expression levels for the corresponding probe sets

**Fig. 5:**
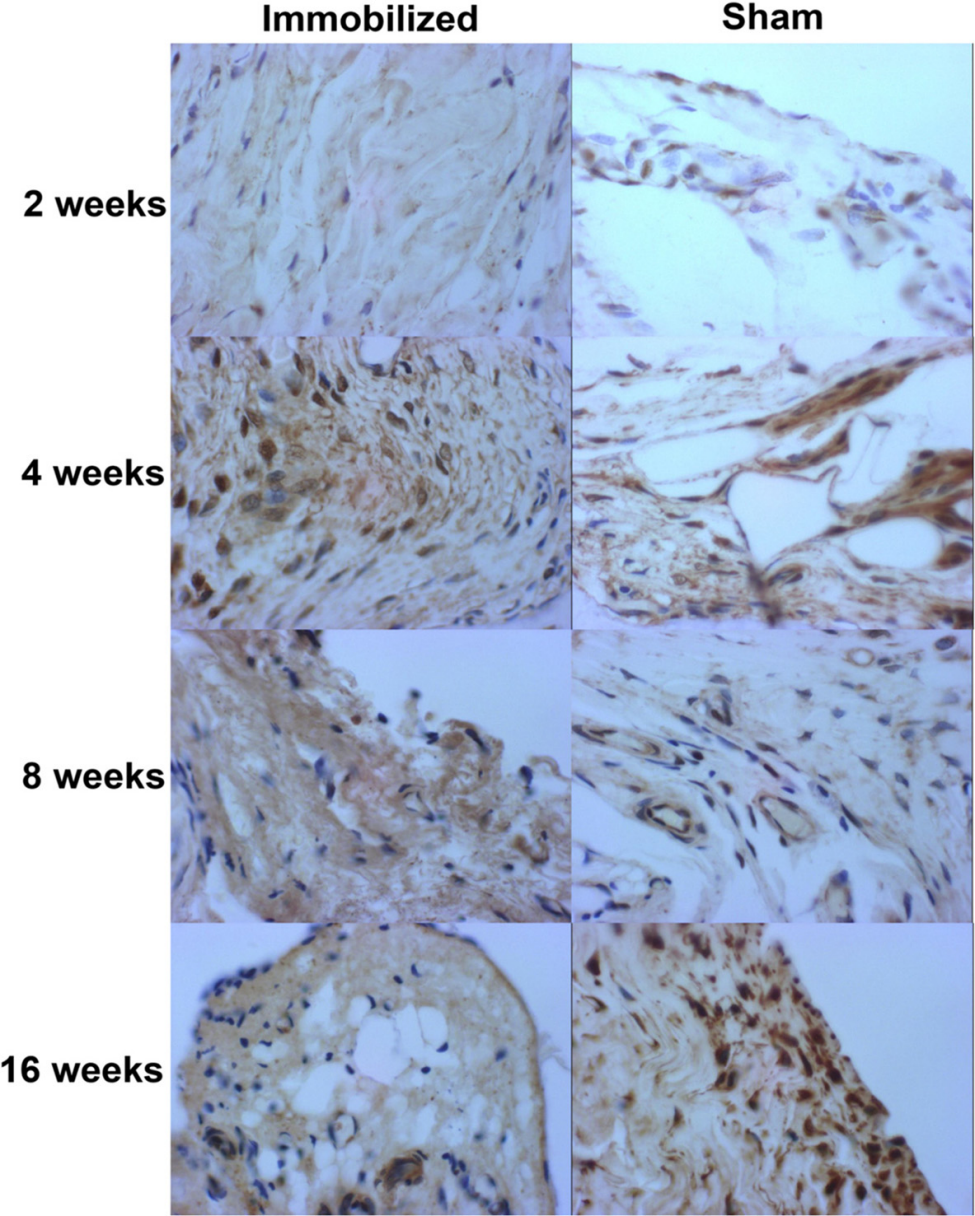
Immunostaining of AGPAT-9 in the posterior capsule of rat knee joints. Haematoxylin staining (blue) and DAB staining (brown) of AGPAT-9 antigen in immobilized and sham-operated rats at time points of 2, 4, 8, and 16 weeks. Cells stained with DAB were considered positively stained. Images were captured with a Marlin F080C digital camera (Allied Vision Technologies) with AVT Smartview 1.5.1 software, magnified to 100X

Three genes enriched in the triglyceride biosynthesis pathway were selected for validation of gene expression changes and included AGPAT-9 (1- acylglycerol-3-phosphate O-acyltransferase 9), P4HB (protein disulfide isomerase is a subunit of the enzyme prolyl 4-hydroxylase) and PCK1 (phosphoenolpyruvate carboxykinase 1). AGPAT-9 protein and transcripts levels varied with a similar profile of decreased expression (Fig. 4 panel b and Fig. 5). The difference in protein staining for AGPAT-9 between sham-operated and immobilized capsule was statistically significant at 2 weeks (p = 0.025). P4HB is a microsomal triglyceride transfer protein involved in intracellular lipid exchange [16] and levels of the corresponding transcript and protein decreased over the time-course (Fig. 4 panel c). Statistically different amounts of protein staining were detected at weeks 8 (p = 0.015) and 16 (p = 0.013). Profiles of expression of PCK1 transcript and protein were different; mRNA levels decreased while protein levels presented a mixed profile of expression over time; low levels were measured at week 2 and 8 with approximately 10 % of cells staining positively and approximately 40 % of cells stained positively at week 4 and 16 (Fig. 4 panel a). The difference in protein staining for PCK1 was statistically significant at 8 weeks (p = 0.0009). Changes in protein levels were not determined at week 1 while gene expression values represent a ratio of expression with week 1 used as a reference.

*P4HB* gene was also enriched in the extracellular matrix synthesis pathway and was involved in the post translational modification of collagens [17]. P4HB transcript and protein levels both decreased over the time-course (Fig. 4 panel c). HSP47 is involved in the folding of collagens [18], and although not included in the list of genes displaying altered expression in association with immobilization, it was selected based on previous published histological data generated from the rat model of knee flexion contractures and reporting an increase of collagen I staining and a decrease of collagen III [19]. Both levels of transcript and staining intensity of HSP47 decreased over the time-course of immobilization-induced knee joint contractures (Fig. 4 panel d). A significant difference in HSP47 protein staining between immobilized and sham-operated capsule was detected at week 4 (p = 0.037).

## Discussion

All immobilized rat knees developed a contracture and structural and genetic changes varied with a similar dynamic. The loss of ROM was gradual during the first 8 weeks of continuous immobilization then a plateau is reached despite extending the immobilization period to 32 weeks [12]. The posterior knee capsule tissue responded to immobilization and deployed a profile of gene expression characterized by time-dependent changes dominated by reduced expression for 92.5 % of genes at week 8. The surface of the articular cartilage became irregular after 2 weeks and progressed rapidly to plateau after 8 weeks [20, 21]. For patients, time spent in the ICU was a risk factor for contractures and a stay of 8 weeks or longer had an odds ratio of 7:1 to develop joint contractures relative to a stay of 2 to 3 weeks [10]. Clinical and experimental observations are indicative of a negative impact of immobilization on the knee joint characterized by changes affecting function, structure and genetic, and with a similar dynamic; an early onset, a progression up to week 8 and a plateau.

Sampling the knee capsule at multiple time points provided a view of the sequence of gene expression events that took place during the process of developing knee flexion contractures. Time-series analysis captured an early increase in gene expression measured at 2 and 4 weeks and a later decrease at week 8. Whether there is a link between early and late events of gene expression measured in the current study remains to be determined. Assessing changes in expression within the immobilization group as opposed to using a paired design in which sham-operated and immobilized are compared at similar time points was strategized to account for the effect of surgery and reduced the heterogeneity of samples.

The integration of the functional analysis results for the 162 immobilization-only genes revealed enrichment of genes involved in lipid synthesis and extracellular matrix pathways. While modulation of expression of genes associated with extracellular matrix pathways was expected in connective tissues such as the knee capsule, the enrichment of genes in the lipid pathways was unexpected [22]. Genes identified as part of triglyceride synthesis all clustered together (cluster 4) and showed a decrease in expression over time. Interestingly, a case of a patient with congenital generalized lipodystrophy type 1 with a mutation in the *AGPAT-2* gene has been reported to have joint contractures [23]. Other inflammatory lipodystrophies and mandibuloacral dysplasia have also been associated with joint contractures [24]. While a decrease in triglycerides has been shown to result in adipocyte lipotoxicity, mitochondrial dysfunction and oxidative stress [25], a potential causative role of triglyceride pathways in joint contractures has yet to be investigated.

The immobilization-only genes included collagen genes *Col2a1, Col10a1*, and *Col11a1,* all previously associated with human genetic disorders with altered joint mobility [26–31]. Col11a1, a fibrillar collagen, had a profile of expression that decreased over time while Col2a1 and Col10a1, both found in hyaline cartilage, increased in early time points and decreased at later time points. The role of those small collagens in capsule elasticity remains to be discovered. The principal collagens in the capsule, collagen types I and III, are influenced by immobilization; an increase in collagen I and a decrease in collagen III staining were previously documented [19]. In the current study, the transcripts for both Col1 (Col1a1 and Col1a2) and Col3 (Col3a1) were expressed at very high levels at week 2 and 4, possibly as an effort to remodel the capsule extra-cellular matrix, and reduced at week 8 and 16 when the contracture became chronic. Changes in mRNA levels parallel the protein changes for collagen III but not for collagen I. The regulation of collagen synthesis and maturation was not limited to transcriptional regulation but also modulated by the activity of the molecular chaperone Hsp47 [18]. Levels of Hsp47 mRNA and proteins both decreased at week 8 and 16 in samples from immobilized knee joints. A role for collagen associated protein in the establishment of capsule contractures was supported by the identification of chondroadherin (CHAD) in the cluster analysis. CHAD is a cell-binding protein found in cartilage and other tissues and is known to strongly associate with collagen, specifically collagen type II [32, 33].

Several extra-cellular matrix degrading metalloproteinases were also enriched in the immobilization-only gene list: MMP3, MMP9, and MMP13. MMP9, which degrades type IV and type V collagens, showed an increase in expression over time [34, 35]. MMP3 and MMP13, which can combine to degrade a wide variety of extracellular matrix proteins, showed a decrease in expression over the time course [36–39]. Cadherin-1 and cadherin-2, both up regulated over time in the capsule of immobilized knee joints, have important roles in cell adhesion [40, 41]. Enhanced cell adhesion mechanisms could explain the reduced synovial intima length resulting when the folds of the posterior capsule, relaxed when the knee joint is immobilized in flexion, adhere to each other and fuse, and leading to mechanical limitation [15, 42].

More studies are required to demonstrate the causal role of the identified genes and pathways in the limitation of knee ROM induced by immobility over time. Previous work to characterize the pathophysiology of knee flexion contractures includes the examination of sagittal rat knee sections prepared from immobilized and sham operated rat knees using light microscopy. Changes include the presence of surface irregularities of the articular cartilage, a reduced number of chondrocytes and reduced collagen matrix staining [20, 21]. In the knee posterior capsule, a decreased number of proliferating synoviocytes and increased intima adhesions were observed [15, 42]. In the current study, the manifestations of joint immobility were studied at the molecular level and changes in gene expression in the posterior capsule are described.

The role of mechanical stimulation in joint homeostasis has been examined in various experimental models designed to reproduce the clinical features of osteoarthritis (OA). Emphasis is often placed on the response to surgical destabilization of the knee to induce OA and analysis of the articular cartilage conducted to interrogate disease pathways [38, 43]. Few studies have considered the contribution of immobilization in the development and progression of OA and the literature is dominated with end-stage OA disease studies. We examined the profile of expression of genes in capsule samples obtained from patients with end-stage knee OA with or without knee flexion contractures and reported differential expression of 4 genes previously associated with fibrosis [44]. The broad spectrum of factors likely to contribute to the differential expression of genes in clinical studies limits the extent of the conclusions and animal studies provide an alternative. Dynamic flexion of the mouse joint and forced flexion/extension exercise applied to destabilized rat joints favored the development of OA [45]. In the mouse model of OA, immobility induced by prolonged anesthesia or by sciatic neurectomy prevented OA up to 12 weeks postsurgery [45]. Microarray analysis of genes expressed in the whole joint of those animals revealed induction of protease genes known to contribute to the degradation of the cartilage matrix that was prevented when joints were immobilized [45]. The authors concluded that immobilization prevented murine OA and the expression of cartilage proteases. The complex nature of animal OA models does not allow for distinguishing between a response to joint damage performed to induce OA and a pure mechanotransductive response. Similarly, whole joints were used to perform microarray analysis and the contribution of individual tissue structures to differential expression could not be established [45]. Our model of immobilization-induced knee flexion contractures and sham operated controls was designed to isolate the joint response to immobilization and previously published results indicate a detrimental role of immobilization on mechanical function, cartilage structure and capsule elasticity. The cartilage response to immobilization was indicative of degeneration; loss of chondrocytes, decreased collagen matrix staining and irregularity of the superficial layer all supporting a degenerative process triggered by the absence of mechanical stimulation [20, 21].

There are limitations to the microarray analysis of the posterior knee joint capsule in the study of joint contractures. In this study, we focused our analysis on the capsule and were not able to determine the role that other soft tissues might have in joint contractures. The posterior knee capsule is composed of different types of cells, mainly fibroblasts but also adipocytes, synoviocytes, and endothelial cells all potentially contributing to changes in gene expression. Another limitation is inherent in the microarray experiment itself and the correlation between the levels of mRNA and of protein is tentative at best [46]. Previous studies have reported differences as high as 30 % of the transcripts identified as differentially expressed by microarrays and changes in the corresponding protein levels and have attributed the poor correlation to the limited ability to measure protein levels. The level of protein is likely to be the main contributing factor to biological relevance, and justified the choice of assessing tissue protein levels for validation purposes.

The demonstration of dynamic changes of gene expression in the posterior knee capsule reflects the phenotypic joint response to immobilization over time and provides an opportunity for assessment of within patient changes during the progression of knee contractures as well as treatment response. The profile of capsule gene expression could be used to predict the prevalence of knee flexion contractures and outcome of TKA or of an ICU stay with an opportunity to apply preventive physical therapy prior to the development of irreversible knee flexion contractures in those patients.

## Methods

### Animals

Male adult Dark Agouti rats 12 weeks of age and weighing approximately 325 g (Charles River Laboratories, St-Constant, Quebec, Canada) were kept in an animal-care facility with strict compliance to care and usage protocols approved by the University Ottawa Animal Care Committee. Rats were housed individually in standard cages and maintained under 12- h light/12-h dark conditions at an ambient temperature of 21 °C and fed a standard rat diet (dry food) and water ad libitum.

### Surgical procedures

We immobilized the knee joint of one hind limb of the rats using an internal system implanted surgically as previously described [12]. The knee remained in 135° flexion throughout the duration of the immobilization period and developed a knee flexion contracture [12]. While the anterior joint capsule is stretched, the posterior knee capsule is relaxed and structural changes, with time, will cause biomechanical restrictions. This was the rationale for focusing data collection on the posterior capsule in this model. The control group corresponded to sham operated animals in which only the screws were implanted.

### Experimental design

For each time point: 1, 2, 4, 8, and 16 weeks, we immobilized 8 rats and performed sham surgeries on 8 control animals. At the end of the immobilization period, animals were euthanized and the posterior knee joint capsules of the operated legs were harvested for genome-wide expression and histological analyses.

### RNA isolation

Individual capsules were submerged in 200 μL of ice cold RNAlater® solution and stored at 4 °C overnight, allowing for diffusion of the solution into the tissue. Capsules were transferred into 0.5 ml of ice cold TRIzol®, homogenized, and total RNA was isolated using the manufacturer’s protocol (Invitrogen Life Technology, USA). Extracted RNA was dissolved in 10 μL of RNase free water, treated with DNAse, and stored at −80 °C until all 80 samples were collected. RNA yield and integrity was determined on one μL of extracted RNA using the 2100 BioAnalyzer (Agilent, USA) and the Quant-IT RNA Assay Kit, including pre-diluted RNA standards (Invitrogen Life Technology, USA). For each time point, 8 RNA samples; 4 from the sham operated group and 4 from the immobilized group, were selected based on similar quantity and quality of extracted RNA and used for analysis on arrays.

### cRNA synthesis, labeling and hybridization

Preparation of cRNA probes, hybridization and scanning of arrays were performed at the Genome Quebec facility (Montreal, Quebec, Canada) using the Affymetrix GeneChips® microarray platform and protocols. Total RNA was first reversed transcribed following by RNase H-mediated second-strand cDNA synthesis. The double-stranded cDNA was purified and served as a template for the transcription of biotinylated cRNAs. A second cycle of cDNA synthesis was performed to generate biotinylated cRNA and the amplification factor was 400x. For each harvested knee joint capsule, a biotinylated labeled cRNA sample was hybridized to a single-color array: Rat Genome 230 2.0 Array (Affymetrix), comprising over 31,000 probe sets representing 28,700 well-substantiated rat genes. All probe arrays were hybridized simultaneously for 17 h at 45 °C with constant rotation using an automated system then washed and stained in the Affymetrix Station 400 and scanned by the Affymetrix GeneChip® Scanner 3000 (Affymetrix Technologies). The images from arrays were analyzed to create CEL files.

### Preprocessing of microarray data

The Affymetrix dataset contains data from 40 arrays measuring gene expression in sham-operated and immobilized knee joint capsules harvested at 1, 2, 4, 8 and 16 weeks. Calculations and data extractions from CEL files were performed in the R software environment. For data extraction, “mas5” with target value of 100 and “mas5calls” functions from the “affy” package were used to generate expression values and detection calls respectively. A filtering step was applied to the initial 31099 probe sets to remove probes with detection calls labeled as “absent” across all five time points and corresponding to 5616 probe sets (Fig. 1). For the remaining 25483 probe sets, expression values less than 100 across the five time points, corresponding to 8771 values, were excluded. The expression values of the remaining 16712 probe sets were used to calculate the median of the 5 replicates per probe, at each time point and used for both fold change and temporal expression analyses. The identity of the probe sets was confirmed by comparing the target mRNA sequences on the Affymetrix Rat Genome 230 2.0 GeneChip® with the National Center for Biotechnology Information (NCBI) GenBank database.

### Fold changes in expression, temporal expression and clustering

The two data sets corresponding to time-series gene expression for sham-operated and for immobilized knees, were analyzed independently (Fig. 1). For each series, ratios of expression values of week 2, 4, 8 or 16 over week 1 values were calculated. For analysis of fold changes in expression, median values were used and ratios with a minimum of 1.5 fold change were counted (Fig. 2). For temporal expression analysis, data were log 2 transformed and probe sets with similar profiles of expression were grouped using Cluster Analysis of Gene Expression Dynamics (implemented in the program CAGED: http://dcommon.bu.edu/xmlui/handle/2144/1290) (Fig. 3). CAGED uses a Bayesian model-based clustering algorithm to merge expression profiles into clusters and detects the optimal number of clusters. A polynomial model of order 3 was assumed for analysis and only probes with a fold change in expression higher or lower than 2 fold in at least one time point were considered for functional analysis.

### Functional analysis of candidate genes

The Gene Ontology (GO) enrichment analysis was conducted using the Database for Annotation, Visualization, and Integrated Discovery (DAVID/EASE version 6.7) software [47]. To identify more specific GO terms, analyses were performed using default settings for terms of the GO Biological Processes (BP_FAT), GO Molecular Function categories (MF_FAT) and GO Cellular Component (CC_FAT). Pathways potentially enriched in the list of immobilization candidate genes were identified using the Kyoto Encyclopedia of Genes and Genomes (KEGG) and the Reactome databases of biological pathways [48, 49].

### Human diseases associated with joint contractures or hyper laxity: literature search

Previously reported joint capsule mRNA expression was assigned based on biocuration of the literature for genes and human diseases involving joint mobility and using PubMed and Google Scholar search tools. The biological terms used were the following: joint contractures, joint laxity, joint capsule, fibroblasts and connective tissue, synoviocytes and capsule. A comparison between the immobilization gene expression data generated from the current study and the list of human genes associated with joint contractures or joint hyper laxity was conducted to identify common genes.

### Immunohistochemistry and histological scoring

The altered expression of selected genes was confirmed by immunohistochemistry performed on rat knee sections prepared as previously described [50]. Primary antibodies were diluted in water to the following dilutions: PCK1 (bs5001R, Bioss, Woburn, MA, US) and PLCH (GTX87708, GeneTex, Irvine, CA, US) to 1:750, HSP47 (LS-C137998, LifeSpan Biosciences, Seattle, WA, US) to 1:2000 and P4HB (LS-B3137, LifeSpan Biosciences) to 1:5000. Sections were incubated overnight at 4 °C, except for HSP47 for which sections were incubated for 2 h at 4 °C. Sections stained with P4HB primary antibody were also incubated with MACH 4 Mouse Probe (Biocare Medical). Positive staining was revealed with MACH 4 HRP-Polymer (Biocare Medical) and Liquid DAB Substrate Pack (HK103-5 K, BioGenex, Fremont, CA, US). Sections were counterstained with Hematoxylin (Thermo Electron Corporation, Pittsburgh, PA, US) and mounted. Negative controls corresponded to the omission of the primary antibodies and performing all other steps of the staining protocol. Fields with approximately 50–100 fibroblasts stained positively with DAB were counted as a percentage of total cells by two independent examiners, counts per field were averaged and the field with the largest inter-rater difference per rat was removed. The remaining three fields per rat were plotted and statistical analysis was completed using the R environment. The non-parametric Kruskal-Wallis test was used to determine the statistical difference in protein staining between sham-operated and immobilized capsule at each time point. A p value <0.05 was interpreted as statistically significant.

## Abbreviations

CAGED: Cluster analysis of gene expression dynamics
GO: Gene ontology
KEGG: Kyoto encyclopedia of genes and genomes
OA: Gene
RA: Rheumatoid arthritis
ROM: Range of motion
TKA: Total knee arthroplasthy
